# Microenvironment of pancreatic inflammation: calling for nanotechnology for diagnosis and treatment

**DOI:** 10.1186/s12951-023-02200-x

**Published:** 2023-11-23

**Authors:** Lu Liu, Yiqing Zhang, Xinghui Li, Jun Deng

**Affiliations:** 1 Medical Imaging Key Laboratory of Sichuan Province, Department of Radiology, Affiliated Hospital of North Sichuan Medical College, 1 South Maoyuan Street, Nanchong, 637001 China; 2 Institute of Burn Research Southwest Hospital State Key Lab of Trauma Burn and Combined Injury Chongqing Key Laboratory for Disease Proteomics Army Medical University, Chongqing, 400038 China; 3 Research Center for Tissue Repair and Regeneration Affiliated to the Medical Innovation Research Division and the 4th Medical Center of Chinese PLA General Hospita, PLA Medical College, 28 Fu Xing Road, Beijing, 100853 China

**Keywords:** Nanotechnology, Acute pancreatitis, Targets, Treatment, Diagnosis, Inflammatory microenvironment

## Abstract

Acute pancreatitis (AP) is a common and life-threatening digestive disorder. However, its diagnosis and treatment are still impeded by our limited understanding of its etiology, pathogenesis, and clinical manifestations, as well as by the available detection methods. Fortunately, the progress of microenvironment-targeted nanoplatforms has shown their remarkable potential to change the status quo. The pancreatic inflammatory microenvironment is typically characterized by low pH, abundant reactive oxygen species (ROS) and enzymes, overproduction of inflammatory cells, and hypoxia, which exacerbate the pathological development of AP but also provide potential targeting sites for nanoagents to achieve early diagnosis and treatment. This review elaborates the various potential targets of the inflammatory microenvironment of AP and summarizes in detail the prospects for the development and application of functional nanomaterials for specific targets. Additionally, it presents the challenges and future trends to develop multifunctional targeted nanomaterials for the early diagnosis and effective treatment of AP, providing a valuable reference for future research.

**Graphical Abstract:**

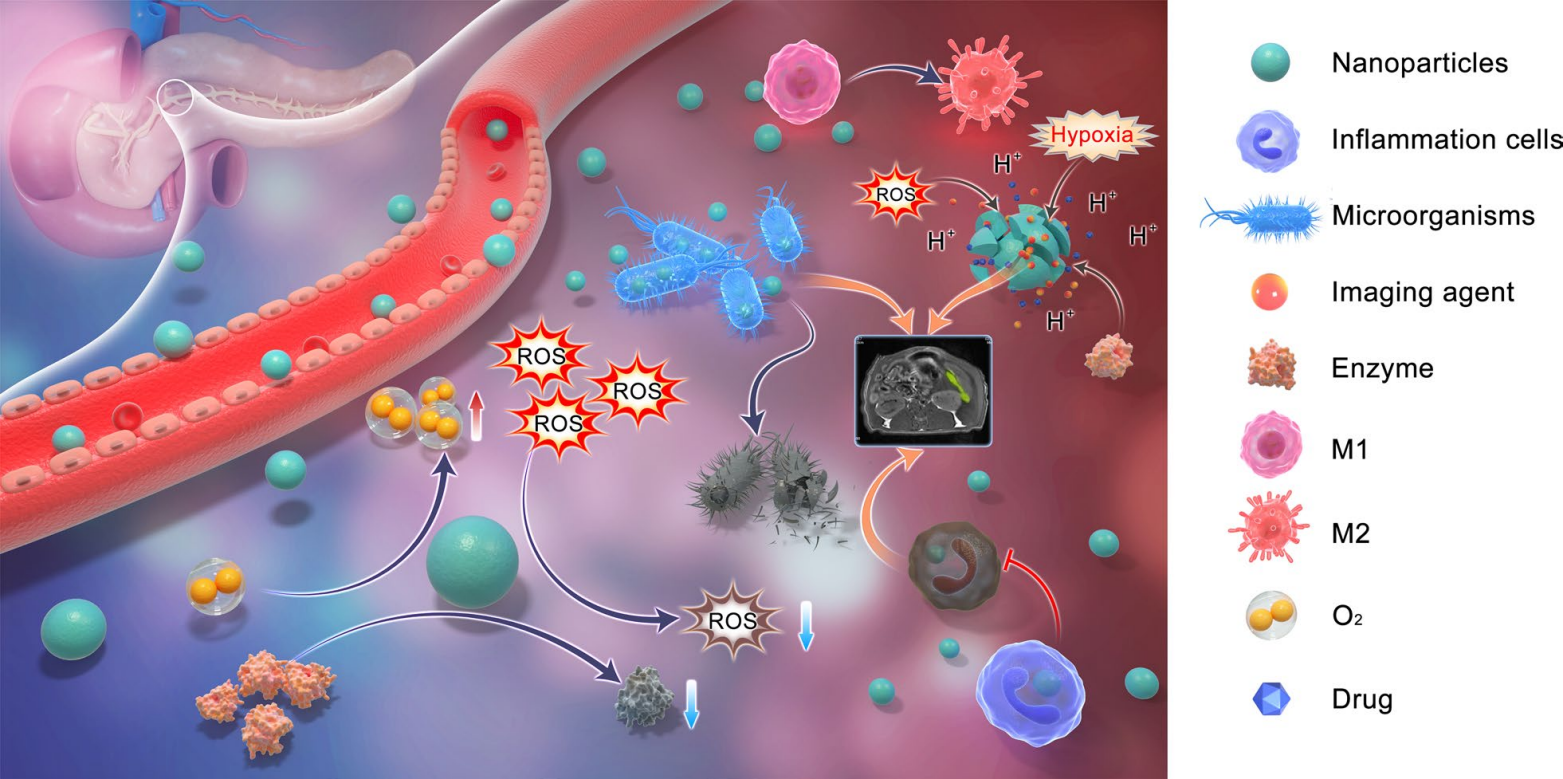

## Introduction

Acute pancreatitis (AP) is a potentially fatal disease with high morbidity and mortality[1]. The typical clinical symptom is persistent severe pain in the epigastrium with abdominal distension, nausea and vomiting. In recent years, its incidence has increased over time [2, 3]. Gallstones and alcohol are common causes of AP [4], leading to activation of trypsinogen, which further activates other digestive enzymes and causes self-digestion in the pancreas [5]. In terms of the course of the disease, it progresses rapidly and the patient develops moderate AP (MAP) or severe AP (SAP), resulting in infectious necrosis, systemic inflammatory response syndrome (SIRS), and multiple organ dysfunction syndrome (MODS), with a mortality rate of 20–40% [6, 7]. Currently, the diagnosis of AP mainly relies on laboratory tests and imaging examinations, which have lower sensitivity for detecting early AP, leading to a decrease in the diagnostic rate of AP and aggravation of the disease. In terms of treatment, general therapy for AP includes close monitoring of vital signs, fluid balance, pain relief, nutritional support, and infection prevention [7, 8]. However, these methods usually fail to suppress the early response of SIRS and prevent subsequent organ failure, and no effective treatment for AP is currently available [9]. Thus, there is an urgent need for a new strategy for the diagnosis and treatment of AP.

The occurrence of AP is a complex, multifactorial, pathophysiological process. Pathological calcium signaling, mitochondrial dysfunction, impaired unfolded protein response, endoplasmic reticulum (ER) stress, and impaired autophagy are among the multiple factors contributing to the pathophysiological changes in the pancreas [5, 10]. The main pathological changes in the pancreas include the activation of trypsin, aggregation of inflammatory cells, and the excessive release of proinflammatory factors and reactive oxygen species (ROS), along with other factors, producing an abnormal microenvironment. These in turn result in a systemic inflammatory response and extensive pancreatic injury. Therefore, the identification and regulation of relevant indicators of the inflammatory microenvironment may be the key to diagnosing or treating pancreatitis.

In recent years, researchers have made remarkable progress in both the diagnosis and the treatment of AP by developing highly sensitive diagnostic tools and drugs targeting microenvironmental changes. Among these approaches, nanotechnology has attracted widespread attention because of its advantages of high sensitivity, specificity, multimeasurement ability, and targeted therapy [11]. Specifically, nanotechnology can target indicators in the microenvironment for the diagnosis and modulation of a disease. For example, Cheng et al. designed an MMP-13/pH-responsive nanoprobe (CMFn@HCQ) for the diagnosis and treatment of inflammation [12]. It was also reported that ferritin nanocages (CMFn) can be used for fluorescence imaging in response to the overexpression of metalloproteinases (MMP-13), a group of protein hydrolases that are related to the degree of inflammation in the microenvironment. It was also shown that the CMFn@HCQ nanocages could release hydroxychloroquine (HCQ) continuously into an acidic microenvironment, which significantly reduced local inflammation. ROS, as free radicals, are closely related to inflammation. The imaging and regulation of ROS can realize the early diagnosis and treatment of AP. Our group also developed a novel nanotheranostic agent (named TMSN@PM) with the ability to target inflammatory sites. Under acidic conditions featuring excessive ROS, TMSN@PM was shown to degrade and release manganese ions for magnetic resonance imaging (MRI) to assess the severity of inflammation. It was found that the T_1_-weighted signal was enhanced in the pancreatic region, which peaked 3 h after TMSN@PM injection. TMSN@PM also scavenges excess ROS and reduces JNK and hypoxia-inducible factor-1α (HIF-1α) activation, thereby reducing inflammation. Compared with the findings in an untreated group, ROS in the pancreas decreased significantly after TMSN@PM treatment, which attenuated the damage to pancreatic tissue [13].

As mentioned above, the occurrence and development of AP are determined by changes in the microenvironment. Significant progress has been made in the early detection and treatment of AP by nanoscientists who have proposed various effective strategies targeting indicators in the inflammatory microenvironment in AP. This review summarizes the application of nanotechnology in modulating indicators related to the inflammatory microenvironment of AP for the early diagnosis and treatment of this disease, which has not been reviewed before. It also discusses the advantages and prospects of nanomaterials in the clinical diagnosis and treatment of AP as well as highlight the limitations of research performed to date to provide new ideas for future development directions.

## Pathogenesis and inflammatory microenvironment of AP

**Fig. 1:**
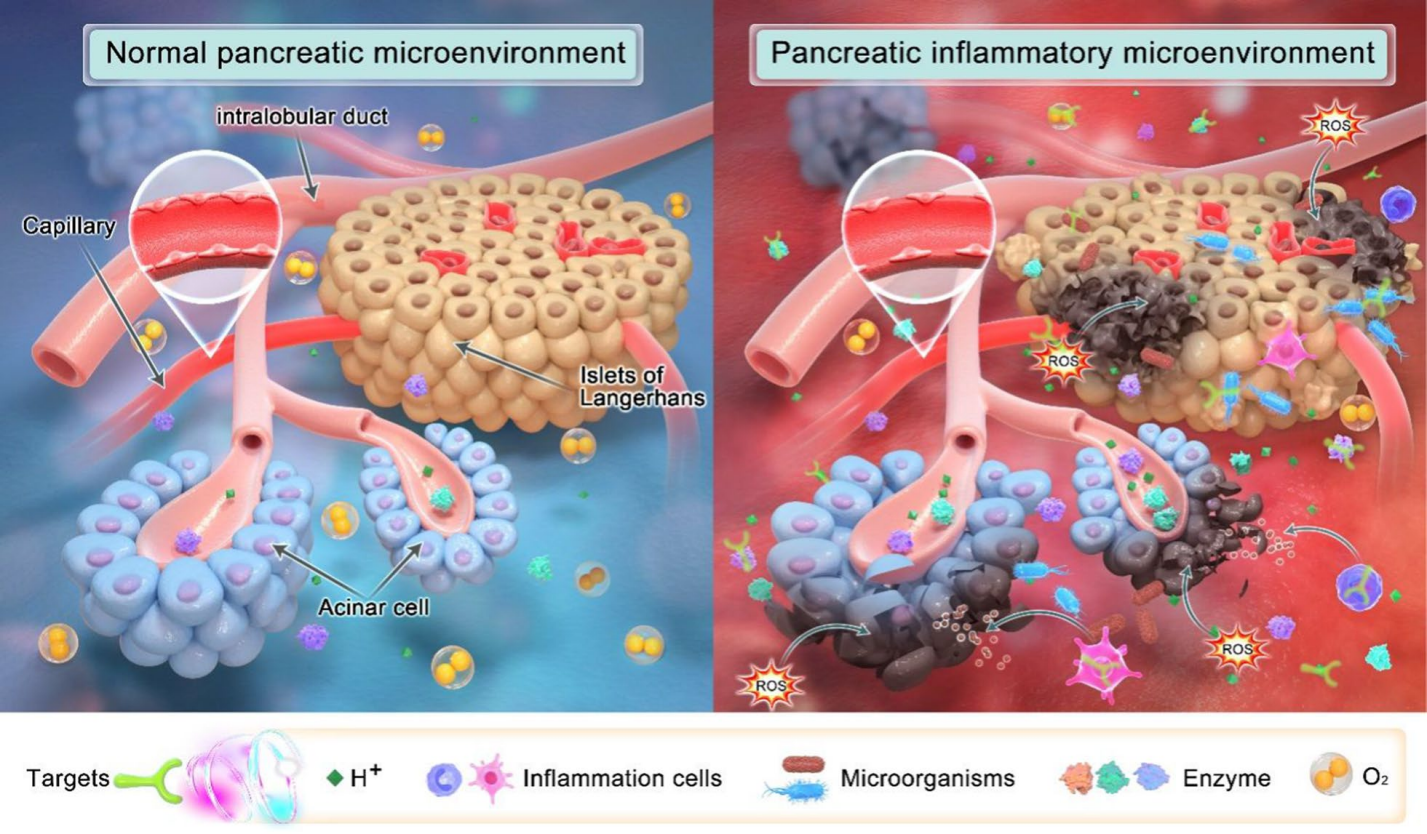
Schematic diagram of microenvironmental targets of pancreatic inflammation. Compared with normal pancreas, AP is characterized by tissue hypoxia and increases in ROS, enzymes, inflammatory cells, H^+^, and microorganisms, creating an inflammatory microenvironment that exacerbates the pancreatic dysfunction. Therefore, the formation of an inflammatory microenvironment in AP is a potential target for imaging and therapy with the application of nanomaterials

Factors such as trypsinogen activation, calcium overload, and mitochondrial dysfunction contribute to AP’s complex pathogenesis, and its diverse etiology and clinical manifestations make its traditional diagnosis and treatment difficult. In the pathophysiology of AP, the activation of trypsin is an early intra-acinar event, which leads to the activation of other digestive proteases and early pancreatic injury [5]. Activation of inflammatory cells such as macrophages releases proinflammatory cytokines that exacerbate the progression of pancreatic inflammation [14]. The excessive release of ROS further worsens the pancreatic cell injury [15], producing various damage-related molecules. Multiple signaling pathways including nuclear factor-kappa B (NF-κB) and toll-like receptor (TLR) are activated, triggering an inflammatory cascade response [16, 17]. Additionally, the impairment of pancreatic cell function and the strengthening of glycolysis lead to the decrease of pH. As shown in Fig. 1, in the pathophysiological process of AP, a large number of substances (e.g., H^+^, digestive enzymes, ROS, and inflammatory cells) are overexpressed and accumulate in the inflammatory site to form an inflammatory microenvironment that has been demonstrated to be significant in disease development [18].

## Nanotechnology-based diagnosis and treatment of AP

With the rapid development of nanotechnology, the emergence of integrated nanoprobes for early diagnosis, drug delivery, and targeted therapy has provided a promising approach for the diagnosis and treatment of AP. Recently nanotechnology-based theranostic strategies for the inflammatory microenvironment have been proposed. Aiming at the abnormal indicators in the microenvironment, nanotechnology has led to new theranostic methods for AP by preparing functional nanocarriers and nanoscale drugs.

Nanotechnology refers to science, engineering, and technology conducted at the nanoscale (from 0.1 nm to hundreds of nanometers), in which nanomaterials are made multifunctional by surface modification, encapsulation and controlled release, and the modulation of physical properties [19–21]. It is widely used in drug delivery and development [22, 23], imaging [24], anticoagulation and hemostasis [25], phototherapy [24], immunotherapy [26, 27], and multimodal combination therapy [28]. Nanomaterials including inorganic nanoparticles, liposomes, micelles, and dendrimers have been widely reported to exhibit excellent biocompatibility, biodegradability, non-toxicity, and various beneficial physical properties as drug carriers [29–32].

In terms of diagnosis and treatment of inflammatory diseases, nanomaterials are characterized by their small size and large specific surface area. Nanodrugs and nanocarriers can enhance the bioavailability and effectiveness of drugs by targeting aggregation at inflammatory sites through the effect of extravasation through leaky vasculature and subsequent inflammatory cell-mediated sequestration (ELVIS effect) [33]. Besides, nanocarriers can improve the pharmacokinetics of drugs in the microenvironment or at the cellular level by protecting drugs from degradation, crossing biological barriers, prolonging drug half-life, and targeted controlled release, so as to improve the therapeutic effect [34–37]. For example, Chuang et al. developed a nanocarrier system that passively targets intestinal M cells, markedly improving the water solubility of curcumin (CUR) and improving the recovery of AP [38]. Owing to the advantages offered by nanotechnology, it is now widely used in diagnosing and treating inflammatory diseases, including AP.

Multiple etiologies contribute to the occurrence of AP, which is characterized by pancreatic inflammation. In the inflamed pancreas, large numbers of ROS, inflammatory cells, enzymes, H^+^, and other substances form an inflammatory microenvironment that can exacerbate AP progression through various mechanisms. In recent years, a series of drug delivery systems and nanodrugs have been designed based on the microenvironment specific to inflammation to achieve the diagnosis and treatment of inflammatory diseases, including AP. The diagnosis and treatment of AP based on different indicators in the inflammatory microenvironment by using nanotechnology are described below.

### Inflammatory cells

There are two main inflammatory cells related to the inflammatory response: macrophages and neutrophils [39]. Neutrophils are the most abundant white blood cells in the human body, which play a vital role in acute inflammation [40]. In the early stage of inflammation, neutrophils are activated and rapidly reach the site of inflammation to eliminate pathogens [41]. Macrophages, as innate immune cells, are involved in cytokine release, tissue damage and repair, and immune disorders [42]. However, excessive activation of macrophages and neutrophils can lead to tissue damage and an inflammatory cascade response, which can result in inflammatory diseases. In the pathogenesis of AP, macrophages and neutrophils have emerged as potential targets in the diagnosis of AP and in the control of systemic inflammatory responses and complications.

As a member of the innate immune system, macrophages play a key role in the recognition of pathogens and defense against them via phagocytosis. Nanomaterials are small in size, so macrophages can internalize nanocontrast agents for the imaging of tissues and organs [43]. As a result, researchers have developed various nanomaterials for applications in macrophage imaging, so as to locate the sites of inflammation [44].

**Fig. 2:**
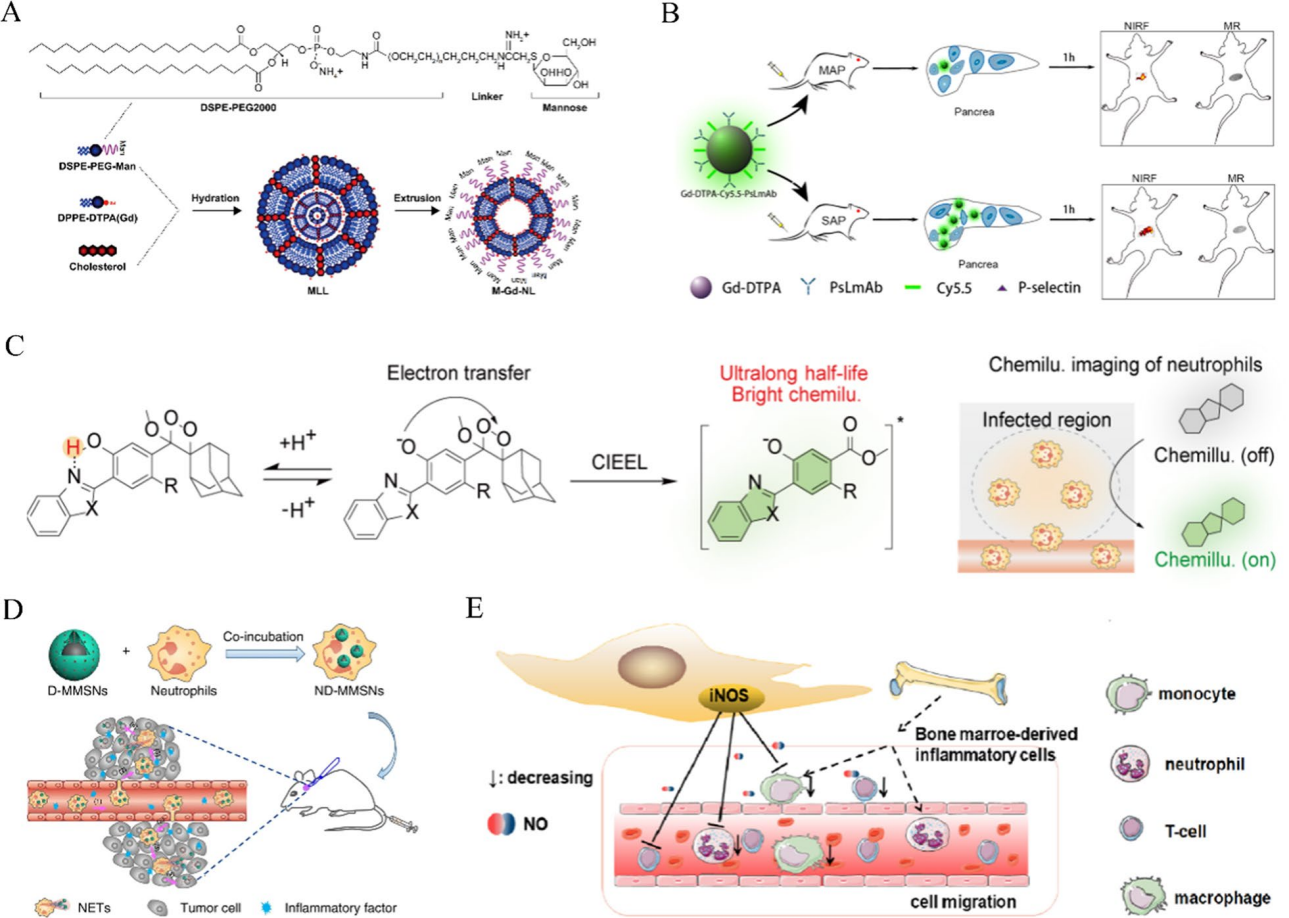
**A** The preparation procedure of Gd-NL and M-Gd-NL based on lipid film method. Reproduced with permission from reference [48]. Copyright 2017, DOVE Medical Press **B** Schematic representation of Gd-DTPA-Cy5.5-PsLmAb for NIRF and MR imaging of MAP and SAP. Reproduced with permission from reference [49]. Copyright 2020, American Chemical Society **C** Schematic illustration of the mechanism of activatable chemiluminescent probes. Reproduced with permission from reference [62]. Copyright 2022, John Wiley and Sons **D** Fabrication and targeted-therapeutic schematics of ND-MMSNs. Reproduced with permission from reference [63]. Copyright 2018, Springer Nature **E** Schematic illustration of nanoparticle-encapsulated CQ/TAM combined with MSCs for arresting the increasing severity of AP in mice through iNOS (IDO) signaling. Reproduced with permission from reference [68]. Copyright 2022, Elsevier

MRI is a common clinical diagnostic modality, which has been used as an important diagnostic tool for AP given its advantages of high soft-tissue contrast, high spatial resolution, and low ionizing radiation. The most common method to diagnose AP with an MRI scanner is to image superparamagnetic iron oxide nanoparticles (SPION). In vivo, SPION can be taken up by macrophages and become a macrophage marker, helping to detect damage to the pancreas and related organs at an early disease stage [45]. Nanotechnology can improve the capabilities of MRI by developing macrophage-targeted-accumulation contrast agents [46]. As reported previously, a novel Gd-containing contrast agent named Gd(III)-dithiolane gold nanoparticles can be phagocytosed by macrophages for targeted accumulation in the pancreas, which showed a very high r_1_ relaxation rate at both low and high magnetic field strengths for MRI of the pancreas[47]. Decorating nanoparticles with ligands that bind to macrophage surface receptors can improve the targeting of nanoparticles. Since mannose receptors are highly expressed by macrophages, Tian et al. developed novel Gd-DTPA-loaded mannosylated liposomes (named M-Gd-NL) (Fig. 2A). M-Gd-NL can bind to macrophages in a targeted manner in the inflammatory microenvironment and then release Gd-DTPA, resulting in a potent increase in the relaxation rate of Gd-DTPA in macrophages, which substantially enhances the capacity of MRI. This method not only improves the diagnostic capability of MRI, but also enables differentiation between mild and severe AP [48]. Similarly, Long et al. synthesized a P-selectin-targeted, near-infrared fluorescence (NIRF) dye (Cy 5.5)-labeled dual-modal nanoprobe (Gd-DTPA-Cy5.5-PsLmAb) based on the finding that macrophages highly express P-selectin. When PsLmAb of the nanoprobe can bind P-selectin in the microenvironment, the more P-selectin there is, the more Gd-DTPA-Cy5.5-PsLmAb nanoparticles that reside at the site of inflammation, resulting in an enhanced signal in MR/NIRF images (Fig. 2B). This probe can achieve the early diagnosis and treatment of SAP by MR imaging and NIRF imaging, providing a rapid method of visualization for the diagnosis of clinical early-stage SAP [49].

Macrophages also mediate the pathological process of AP through various mechanisms [50, 51]. It has also been reported that macrophages are related to the progression of SAP [52]. During SAP, peritoneal macrophages, alveolar macrophages, and Kupffer cells are activated, which contribute to the damage to various organs. Macrophages can be divided into two subtypes: M1 macrophages and M2 macrophages. M1 macrophages secrete factors related to the proinflammatory stage of AP, while M2 macrophages are mainly involved in pancreatic repair and regeneration [53]. Macrophages can change their phenotype and function spatiotemporally, which is called macrophage polarization. Therefore, regulating the polarization of macrophages is a new direction for the treatment of AP [54, 55]. Kazuaki et al. constructed a nanotechnology-based CO donor (CO-HbV) that can target macrophages and inhibit AP by releasing CO to polarize macrophages toward an M2-like phenotype. CO-HbV was also reported to inhibit neutrophil infiltration in the pancreas and attenuate the subsequent acute lung injury [56]. The degree of severity of AP is related to the number of infiltrating macrophages, which is involved in the development of injuries to the pancreas and multiple other organs. Based on this, researchers have focused particularly on drugs that inhibit macrophage recruitment and deplete macrophages. Tang et al. studied the protective effects of G4.5-COOH and G5-OH on the pancreatic injury of AP mices. It was found that two kinds of dendrimers reduced the inflammatory infiltration of macrophages by inhibiting nuclear translocation of NF-κB in macrophages. Moreover, they also inhibited the expression of proinflammatory cytokines in peritoneal macrophages and significantly decreased the pathological changes of the pancreas [57]. Clodronate liposomes are the most commonly used method to deplete macrophages [58]. Dang et al. loaded liposomes with clodronate and superparamagnetic iron oxide (SPIO), which can be delivered in a targeted manner to macrophages to induce their apoptosis by competing with adenosine triphosphate (ATP), thus inhibiting the release of inflammatory factors and alleviating the renal injury caused by SAP [59]. Different from them, Chen’s team investigated an inflammation-targeted nanoparticle named MU, which was composed of PEG − PLGA and ulinastatin coated by macrophage membrane [60]. In the mouse model of AP, MU can significantly inhibit the secretion of pro-inflammatory cytokines TNF-α and IL-6 by macrophages. In addition, in vitro experiments have proved that MU may play an anti-inflammatory role by reducing the contents of p-IκBα/IκBα and p-p65/p65 through IκBα/NF-κB signaling pathway. Therefore, MU is expected to be an effective targeted drug to inhibit the progress of AP.

Neutrophil infiltration is a hallmark of inflammation. Neutrophils, as a “living” drug delivery carrier, have attracted widespread attention in recent years because of their characteristics of crossing natural barriers, decreasing immune clearance rate and having a long biological half-life [61]. Similar to macrophages, neutrophils can take up nanoparticles [61]. Therefore, researchers have explored various neutrophil tracking probes for disease diagnosis. For example, Huang’s team synthesized three chemiluminescent probes based on benzoazole-phenoxyl-dioxetane for the in vivo imaging of neutrophils in mouse models of peritonitis and psoriasis. These probes activate and prolong chemiluminescence in the presence of neutrophil elastase (NE) (Fig. 2C). In experiments with LPS-induced peritonitis, benzothiazole-phenoxyl-dioxetane (BTPD_NE_) exhibited more intense brightness and a longer half-life than methyl acrylate-phenoxyl-dioxetane (MPD_NE_) [62]. Moreover, Wu et al. developed core–shell structured magnetic mesoporous silica nanoparticles (called MMSNs) and constructed a theranostic platform of ND-MMSNs for internalizing MMSNs loaded with doxorubicin (D-MMSNs) by neutrophils [63]. In the inflammatory mouse glioma model, ND-MMSNs are internalized by neutrophils, which can be targeted to accumulate at the inflammatory site of glioma with chemokines. Then, neutrophils release neutrophil extracellular traps (NETs) and D-MMSNs to realize the diagnosis and treatment of residual tumors (Fig. 2D). Similar to the features of the above diseases, there are a large number of neutrophils in the inflammatory microenvironment of AP, and it is expected that nanoparticles used for neutrophil imaging in the future can be used for the diagnosis of AP.

Neutrophils can release ROS to cause tissue damage [64]. Moreover, the production of NETs can speed up the progression of AP [65]. Neutrophils may serve as a target for the treatment of AP because they can mediate local tissue damage in the pancreas and associated damage to other organs when AP occurs [66]. Nanotechnology provides an plausible pathway for neutrophil-related therapeutic intervention. For example, nucleic acid nanoparticles (tFNAs) were recently reported to regulate cell proliferation and migration, and have potent anti-inflammatory and antiapoptotic abilities against AP. Wang et al. found that compared with a saline group, tFNAs significantly decreased neutrophil activity and alleviated pancreatic injury in a treatment group [67]. Additionally, Liu et al. introduced nanoparticle-encapsulated chloroquine/tamoxifen in combination with bone marrow-derived mesenchymal stem cells (BMSCs) that acted synergistically for the treatment of AP. BMSCs prevented the progression of AP by suppressing the recruitment of neutrophils, macrophages, and CD4^+^ T cells through iNOS signaling (Fig. 2E) [68]. Furthermore, another membrane-encapsulation technology has been applied for targeting inflammation [69, 70]. Membrane-encapsulation technology can confer nanoparticle-derived cell membrane-related functions such as immune evasion [71], crossing barriers [72], and homing to inflammatory sites [73]. Zhou et al. designed neutrophil membrane-coated nanoparticles (NNPs/CLT) that cross the blood-pancreas barrier (BPB), driven to sites of inflammation through chemokine recruitment, which significantly downregulate the level of pancreatic myeloperoxidase and reduce associated lung injuries in AP rats [74].

Macrophages and neutrophils play an important role in the systemic production of inflammatory mediators. Nanodiagnostic and nanotherapeutic agents targeting neutrophils or macrophages can be designed by nanotechnology to assess the severity of AP and suppress overactive inflammatory responses. At present, notable achievements have been made in the research and development of targeted drugs for macrophages and neutrophils. Since macrophages and neutrophils have abundant surface receptors, the development of more nanoparticles targeting these receptors is a promising future strategy.

### Oxidative stress and reactive oxygen and nitrogen species

**Fig. 3:**
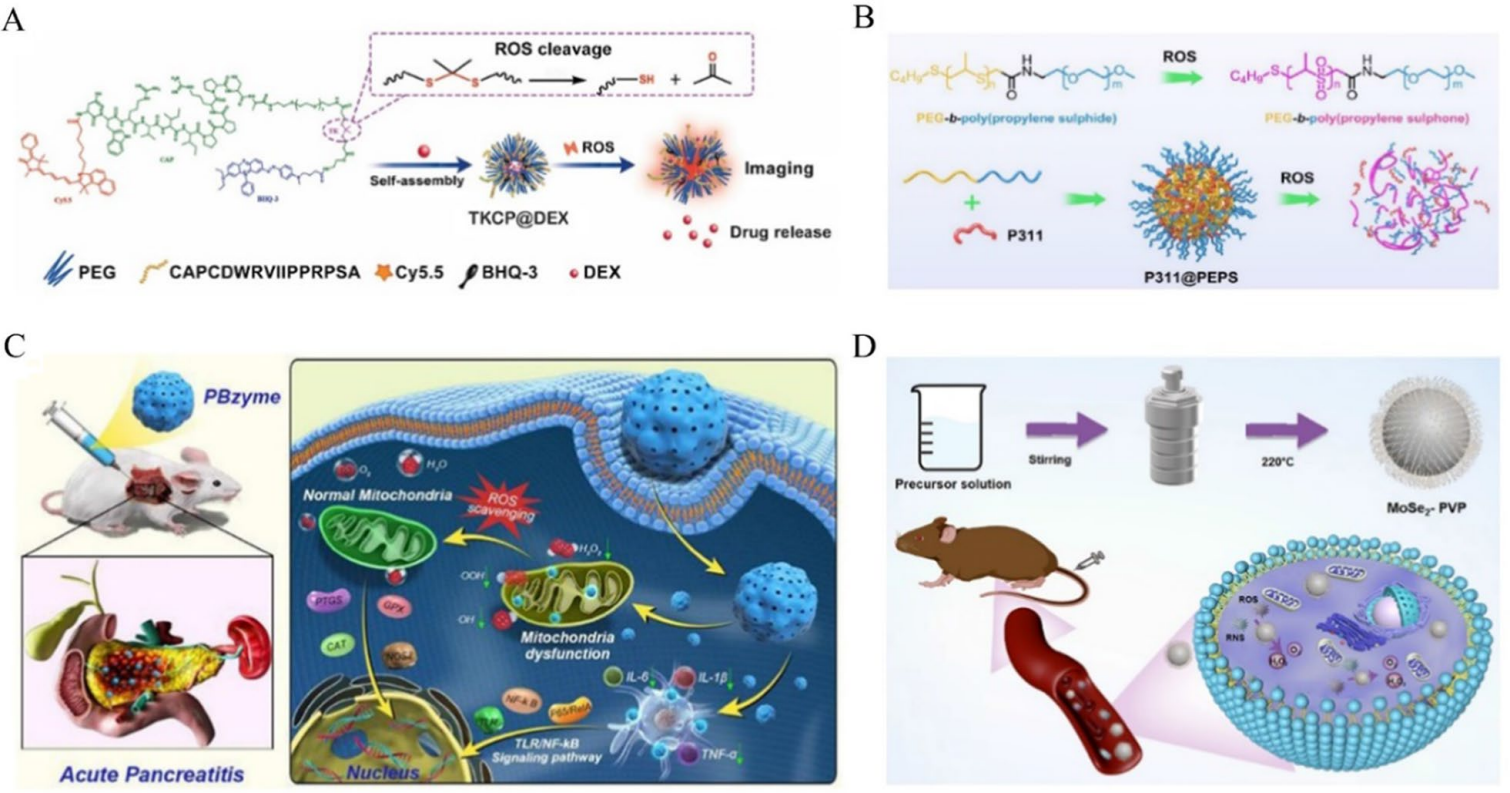
**A** Schematic illustration of the self-assembly of ROS-responsive nanoparticles for bioimaging and targeted therapy. Reproduced with permission from reference [79]. Copyright 2021, BIOMED CENTRAL **B** Schematic illustration of the preparation of poly (ethylene glycol)-block-poly (propylene sulfide) (PEPS) and the therapeutic mechanism of action of ROS-sensitive micelles in vivo. Reproduced with permission from reference [81]. Copyright 2022, Elsevier **C** Schematic illustration of the therapeutic mechanism by which PBzyme prophylactically treats AP by inhibiting activation of the TLR/NF-κB signaling pathway. Reproduced with permission from reference [17]. Copyright 2021, Ivyspring International Publisher **D** Schematic diagram of the steps of synthesis of MoSe2-PVP NPs and the therapeutic mechanism for alleviating AP by scavenging RONS. Reproduced with permission from reference [89]. Copyright 2022, BIOMED CENTRAL

Oxidative stress is an important factor in the progression of AP, and it generates a large number of free radicals including ROS and reactive nitrogen species (RNS), leading to an imbalance between the oxidative and antioxidant systems [75]. ROS, as free radicals, are closely related to inflammation [76]. The existence of ROS not only recruits inflammatory cells to infiltrate and activate inflammatory signal pathways, but also induces oxidative damage, cell apoptosis, and necrosis [77, 78]. In response to ROS, researchers have developed ROS response strategies for the diagnosis and treatment of related diseases. Shen et al. designed theranostic polymeric NPs (named TKCP@DEX nanoprobes) targeting the ROS response in osteoarthritis (OA), which consists of thioketal linkers and cartilage-targeting peptide (TKCP) encapsulated with dexamethasone (DEX) (Fig. 3A). The nanoprobe released Cy5.5 and DEX to target articular cartilage at high levels of ROS, enabling the effective detection and treatment of OA [79]. Similarly, Hong et al. developed ROS-responsive NPs (LFP/PCDPD) for atherosclerosis-targeted diagnosis and bifunctional therapy [80]. LFP/PCDPD released lipid-specific aggregation-induced emission (AIE) fluorescent probe (LFP) and prednisolone in response to ROS and removed lipids, enabling the fluorescent diagnosis and targeted therapy of atherosclerosis. Moreover, our group introduced a therapeutic platform (P311@PEPS) for ROS-responsive micelles to promote the healing of diabetic wounds [81]. P311@PEPS was synthesized by the self-assembly of P311 peptide and ROS-responsive polymer (denoted PEPS) (Fig. 3B). Under conditions with a high level of ROS, P311@PEPS can not only promote cell migration by releasing P311, but also activate the Akt signaling pathway to accelerate the migration of epidermal cells, so as to induce wound re-epithelization. It was also observed that P311@PEPS can improve the bioavailability of P311 by scavenging excessive ROS. Our team also reported a ROS-triggered single-cell nanogel system that scavenges ROS and releases triiodothyronine to induce neural stem cells to differentiate into oligodendrocytes to promote white matter tract regeneration, which showed promising therapeutic effects in white matter after intracerebral hemorrhage [82].

Additionally, functional nanocarriers loaded with antioxidant drugs can treat AP by their targeted aggregation at the site of inflammation. Shahin et al. found that CAPE-loaded nanoliposomes (CAPE-loaded NL) may exert antioxidant, anti-inflammatory, and antiapoptotic effects by modulating Nrf2 and NF-κB signaling. CAPE-loaded NL can reduce myeloperoxidase activity, and TNF-α and caspase-3 expression, thus inhibiting neutrophil infiltration, inflammation, and apoptosis [83]. Li et al. prepared a novel self-nanomicellizing system of empagliflozin (RA-EMP) that addressed the poor water solubility and low bioavailability of empagliflozin (EMP). RA-EMP alleviated the severity of AP by inhibiting oxidative stress and inflammatory factors [84].

Moreover, nanomaterials can be used directly as antioxidants. A series of nanomaterials exerting antioxidant functions have been discovered in recent years, which have significant therapeutic effects on AP. It was reported that inorganic nanoparticles can efficiently scavenge ROS because of their excellent antioxidant capacity. Our group has developed ultrasmall copper-based nanoparticles (Cu_5.4_O USNPs) with enzymatic ROS scavenging capabilities [85, 86]. Compared with other nanomaterials used for treating ROS-related diseases, Cu_5.4_O USNPs exhibit significant antioxidant efficiency at very low doses for many acute and chronic inflammatory diseases. Khurana et al. found that nanoceria showed potent activities mimicking superoxide dismutase and catalase to scavenge free radicals and attenuate cerulein-induced oxidative stress [87]. In the same year, they reported the therapeutic effect of nanoyttria (NY) in AP. NY reduced not only ROS but also the levels of amylase and lipase in plasma. Surprisingly, NY attenuated mitochondrial stress and ER stress through the Nrf2/NF-κB pathway to alleviate experimental AP [88]. Recently, artificial enzymes have drawn attention for their antioxidant capacity mimicking endogenous enzymes. Xie et al. prepared Prussian blue nanoenzyme (PBzyme), which had good preventive therapeutic effects on AP by inhibiting the TLR/NF-κB signaling pathway and scavenging ROS (Fig. 3C). In a cerulein-induced mouse AP model, PBzyme, as an antioxidant, decreased the level of MDA and increased the levels of SOD and GSH, thus alleviating oxidative stress [17]. Xie et al. also developed artificial enzymes (MoSe_2_-PVP NPs) with a one-pot hydrothermal strategy that mimics endogenous antioxidant systems for the treatment of free radical-induced injury. In a cerulein-induced AP mouse model, MoSe_2_-PVP NPs were able to scavenge free radicals to produce strong cytoprotective effects as well as inhibit the release of inflammatory factors, with potent antioxidant and anti-inflammatory effects (Fig. 3D) [89]. Similarly, this group synthesized 2D MoSe_2_@PVP nanosheets (NSs) [90]. MoSe_2_@PVP NSs exhibited thermostable multienzyme activity, which could significantly remove overexpressed ROS and RNS and help to treat diseases related to oxidative stress.

In addition to the use of inorganic nanomaterials with antioxidant properties for the treatment of AP, organic drugs with antioxidant functions can be made directly into nanoparticles. For example, nonmetallic inorganic nanoparticles (Nano-Se) improve pancreatic endocrine and exocrine functions through antioxidant and anti-inflammatory effects [91]. Furthermore, Abizaid’s group prepared cinnamic acid nanoparticles (CA-NPs) to evaluate the therapeutic effect on AP. CA-NPs reduced the MDA level and downregulated caspase-3 expression by inhibiting various signaling pathways, thereby reducing oxidative stress, inflammation, and apoptosis [92].

Nanotechnology-based delivery systems not only prevent the pathological progress of pancreatitis by inhibiting oxidative stress and acinar cell damage, but also inhibit ROS-related inflammatory signaling pathways to play an anti-inflammatory role, thus reducing various types of inflammation. Furthermore, nanocarriers can release imaging agents in the microenvironment in response to ROS, improving the diagnosis of diseases. Similar to previously mentioned inflammatory conditions, ROS are overexpressed in the inflammatory microenvironment of AP, which provide a potential target to develop ROS-responsive nanocarriers for the diagnosis and treatment of this disease. These results suggest that ROS in the inflammatory microenvironment can be an important target for the diagnosis and treatment of AP.

### Enzymes

The premature activation of trypsin leads to the activation of other pancreatic enzymes when AP occurs, thus increasing the severity of pancreatitis [5, 93]. Therefore, there are numerous digestive enzymes in the AP inflammatory microenvironment and enzymes excessively released by the pancreas can be detected and used as candidate targets for AP diagnosis [94]. However, some of the conventional methods are aimed at determining the concentrations of enzymes in peripheral blood and cannot overcome the limitations caused by the lipid-water interface in lipase assays, making them less sensitive.

**Fig. 4:**
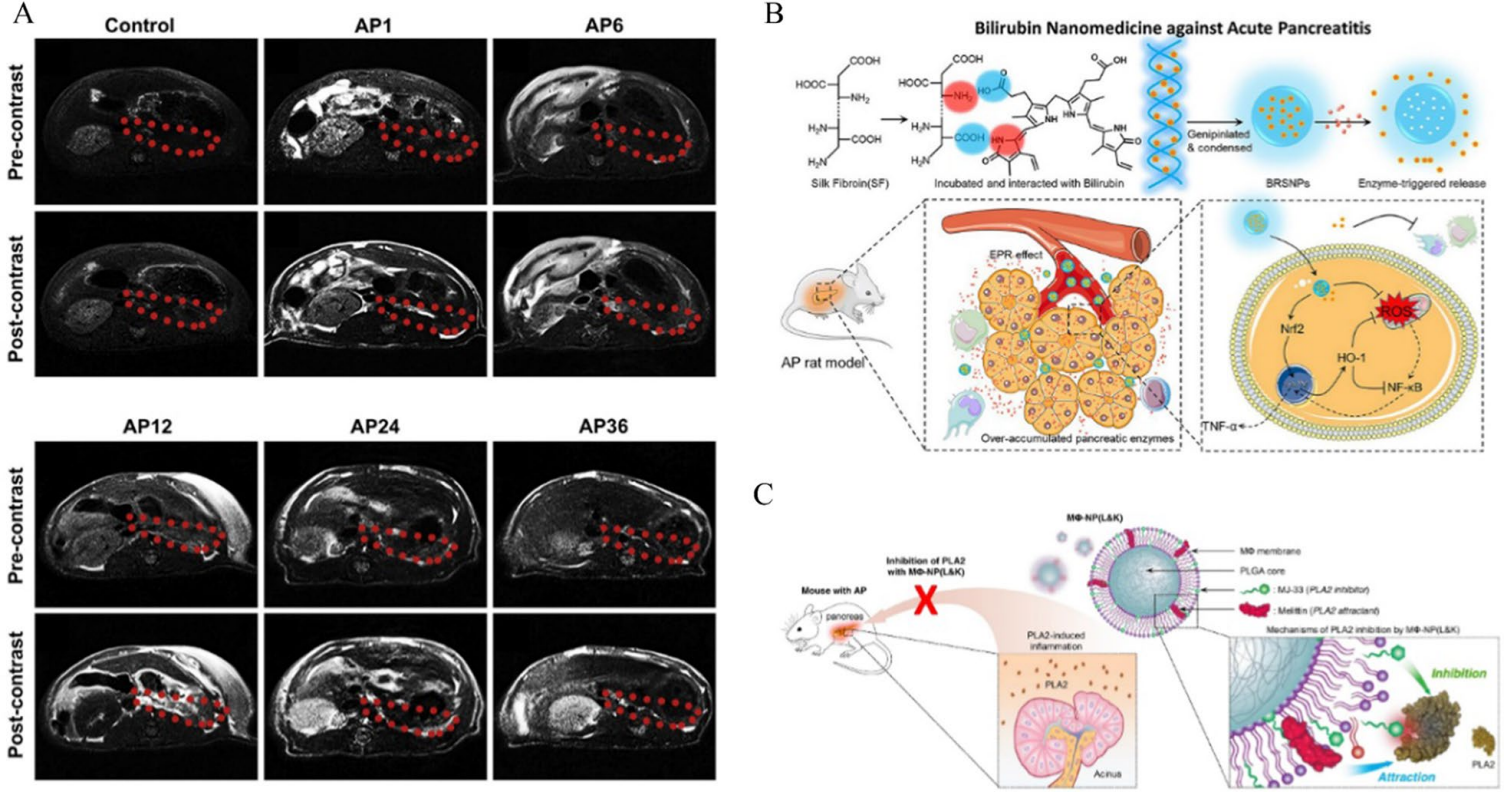
**A** Representative MRI of SD rats before and after the tail vein injection of Gd-DTPA-FA. Reproduced with permission from reference [96]. Copyright 2014, Elsevier **B** Schematic graph of bilirubin loaded silk fibroin nanoparticles (BRSNPs) for the experimental AP application. Reproduced with permission from reference [97]. Copyright 2020, Elsevier **C** Schematic representation of MΦ-NP(L&K) designed to inhibit PLA2 during AP progression. Reproduced with permission from reference [99]. Copyright 2021, Springer Nature

In response to the shortcomings of traditional detection methods, nanomaterials make it possible to substantially improve the affinity between the probe and the enzyme and the interfacial catalytic efficiency, thus realizing lipase detection with higher sensitivity and a lower detection limit with the help of the AIE mechanism [95]. Furthermore, enzyme-responsive nanocarriers can be constructed for drug delivery based on the characteristics of AP for diagnosis and even treatment. Zhang et al. synthesized a conditionally activated, gadolinium-containing MRI nanoprobe (named Gd-DTPA-FA) via the conjugation of DTPA-FA ligand and gadolinium acetate. This probe became soluble when conditionally activated by lipase activity, which increased T_1_ and enhanced MRI signal intensity in early AP. It was found that the signal intensity of the position corresponding to the pancreas in AP mices was significantly higher than that in the control group on T_1_-weighted images after the intravenous injection of Gd-DTPA-FA nanoparticles, and the highest signal intensity was observed at 6 h (Fig. 4A). These results show that Gd-DTPA-FA can be used for MR imaging of early AP [96]. Furthermore, Yao et al. designed targeted bilirubin-loaded silk fibrin nanoparticles able to selectively deliver to inflammatory lesions in the pancreas and release bilirubin rapidly in an enzymatic reaction to exert antioxidant and anti-inflammatory effects, which significantly reduce the damage to the pancreas and related tissues and organs (Fig. 4B) [97].

Surprisingly, nanocarriers can also carry drugs to directly inhibit the release and activity of enzymes. At present, somatostatin is often used clinically to inhibit the release of pancreatic enzymes. However, its short half-life affects the therapeutic effect. Cervin et al. found that the combination of lipid-based liquid crystalline nanoparticle carrier and somatostatin (SST) can prolong the circulating half-life of SST, which shows remarkable potential for treating AP [98]. It has also been reported that phospholipase A2 (PLA2) is a pathogenic factor of AP, which damages acinar cells and promotes disease progression. Bionic nanotechnology provides another therapeutic direction to treat AP. Recently, Zhang et al. obtained macrophage membrane-coated nanoparticles [MΦ-NP(L&K)] by doping melittin and MJ-33 into macrophage membrane-coated nanoparticles. MΦ-NP(L&K) lures and kills serum PLA2 by leveraging the function of the macrophage membrane, which reduces the level of proinflammatory cytokines (Fig. 4C). In mouse models of mild and severe AP, MΦ-NP(L&K) significantly attenuated alveolar necrosis or immune infiltration and effectively reduced the severity of AP[99].

As biomarkers, digestive enzymes can be used to diagnose AP. Moreover, as important components of the inflammatory microenvironment, they are important to the development of AP and used to regulate the inflammatory microenvironment. Functional composite nanoparticles have multiple roles in the diagnosis and treatment of AP. First, as drug carriers, they can prolong the half-life of drugs; second, they can react with digestive enzymes in the microenvironment to release drugs; finally, they can bind with enzymes covalently or noncovalently to inhibit enzyme activity. Therefore, the design of functional composite nanoparticles targeting the inflammatory microenvironment using the biocatalytic properties of enzymes is extremely promising.

### pH

The decrease of pH is one of the characteristics of the inflammatory microenvironment. When AP occurs, enhanced glycolysis of inflamed tissue leads to increased lactate production and a decrease in pH. Impaired endocrine and/or exocrine function of the pancreas in AP patients inhibits bicarbonate secretion by ductal cells, leading to enhanced acidification of the acinar luminal space. Lowering of pH promotes trypsinogen activation by cathepsin B [100], leading to self-digestion of the pancreas. Furthermore, persistent extracellular acidification can disrupt cell junctions and lead to the leakage of zymogen into the interstitial fluid [101]. Thus, extracellular acidification exacerbates the development of AP.

**Fig. 5:**
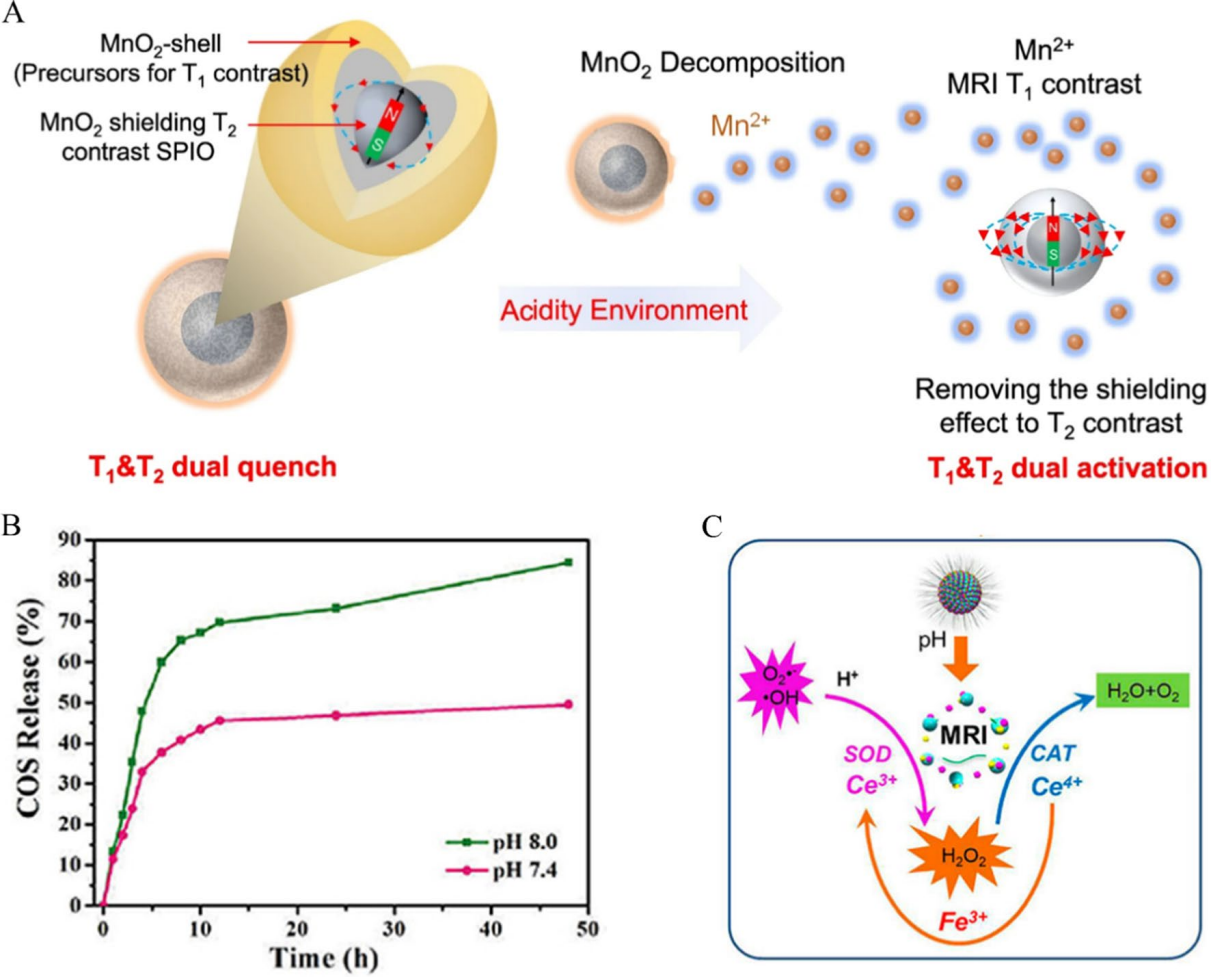
**A** SPIO@SiO_2_@MnO_2_ shows weak T_1_ and T_2_ contrast intensity in normal physiological conditions, as the T_2_ signal of SPIO is quenched by the MnO_2_ layer. In the acidic environment of a tumor or inflamed tissue, the MnO_2_ layer will decompose into magnetically active Mn^2+^ (T1-weighted), and the T_1_ and T_2_ signals are sequentially recovered. Reproduced with permission from reference [104]. Copyright 2022, Springer Nature **B** The cumulative release of COSs from COS@SiO_2_ at pH 7.4 and pH 8.0. Reproduced with permission from reference [105]. Copyright 2020, Frontiers Media S.A. **C** Schematic illustration of biodegradation, ROS scavenging effects, and enhanced theranostic functions by Fe/Ce-MSN-PEG NPs. Reproduced with permission from reference [108]. Copyright 2022, Frontiers Media S.A

In recent years, pH-responsive drug carriers have achieved good results in the diagnosis and treatment of various diseases including AP by targeting drugs to sites of inflammation and modulating drug release in response to pH stimuli [102, 103]. In terms of diagnosis, Lu et al. synthesized a pH-responsive MRI contrast agent, SPIO@SiO_2_@MnO_2_, which can improve the diagnostic accuracy of MRI in an acidic environment by decomposing manganese dioxide (MnO_2_) into Mn^2+^ to increase T_1_- and T_2_-weighted signals (Fig. 5A) [104]. Experimental results demonstrated that the contrast sensitivity of diseased tissues is about 12.3 times that of normal tissues. As for treatment, Mei et al. developed porous COS@SiO_2_ nanocomposites that enable the continuous release COSs and maintain the drug at high concentrations in a pH-controlled manner, which helps to reduce the severity of SAP and its associated lung injury [105]. It was found that the release rate of COS was greater at pH 7.4 than at pH 8.0 (Fig. 5B). Yang’s team used chloroquine diphosphate (CQ) for gene transfection to construct Ca-CQ-pDNA-PLGA-NPs that can deliver targeted genes to the site of pancreatitis and protect the pancreas from deterioration based on pH changes. Compared with the findings at pH 7.4 and pH 6.8, the cumulative pDNA release at pH 4.5 exceeded 30% within 24 h and eventually reached 60% within 4 weeks [106]. Similarly, Hassanzadeh et al. prepared a neutrophil membrane-encapsulated nanoformulation (FA-SF-NPs) using silk fibroin (SF) and ferulic acid (FA). FA-SF-NPs released FA with higher kinetics in a low-pH environment compared with the findings at physiological pH, thereby downregulating serum enzymes and oxidative stress-related indicators to reduce the severity of AP [107]. Moreover, with the development of nanotechnology, pH-responsive theranostic nanoplatforms have been successively developed. Dou et al. constructed metal Fe/Ce-doped mesoporous silica nanoparticles (Fe-Ce-MSN) for the treatment of inflammation and oxidative stress-related diseases [108]. In the mildly acidic environment of inflammatory sites, Fe-/Ce-MSN nanoparticles released Fe ions, which enhanced the T_2_-weighted signals. Additionally, Fe-Ce-MSN NPs not only scavenged overproduced ROS but also controlled the production of tumor necrosis factor-α (TNF-α) and interleukin-1β (IL-1β), with significant antioxidant and anti-inflammatory effects (Fig. 5C).

The inflammatory response of AP fosters a pH gradient between inflamed and healthy tissues, which provides a suitable physiological stimulus for pH-responsive drug delivery. pH-responsive drug delivery systems overcome the shortcomings of conventional drug formulations and show advantages in terms of biocompatibility, stability, size, and structural control. Moreover, they can deliver drugs to specific sites in a controlled manner and at predetermined release rates, reducing drug side effects and improving drug efficacy. Therefore, acid-responsive nanocarriers are of high value for the diagnosis and treatment of AP.

As a brief summary, Table 1 shows the strategies of diagnosis and treatment of AP with various nanomaterials.

**Table 1:** Nanotechnology-based strategies for the diagnosis and treatment of acute pancreatitis

Category	Indicators	Nanoagents (Size)	Animal Models	Mechanisms	modes	Refs
Inflammatory cells	Macrophage	Lip-DTPA@AuNP(17.2 ± 2.1 nm) M-Gd-NL (120.2 ± 8.5 nm) Gd-DTPA-Cy5.5-PsLmAb (50 mm) CO-HbV(~ 280 nm) G4.5-COOH, G5-OH (5 nm) SPIO-clodronate-liposomes(100–200 nm) MU(175 nm)	Caerulein and LPS-induced AP L-arginine-induced AP Caerulein-induced AP, L-arginine-induced AP – Caerulein-induced AP Sodium taurocholate-induced SAP Caerulein-induced AP	Gd (III) contrast agents loading of AuNPs and localization to pancreatic tissue for MR imaging Targeting macrophages and increasing T1 Imaging ability P-selectin-targeted MR/NIRF bimodal imaging improves spatial resolution and sensitivity Targeting macrophages and polarizing macrophages toward an M2-like phenotype Inhibition of NF-κB nuclear translocation in macrophages and a reduction in inflammatory cells Selectively inducing macrophage apoptosis and reducing the release of inflammatory mediators Significantly inhibiting the secretion of pro-inflammatory cytokines TNF-α and IL-6 by macrophages	Diagnosis Diagnosis Diagnosis Therapy Therapy Diagnosis and therapy Therapy	[47] [48] [49] [56] [57] [59] [60]
	Neutrophil	tFNAs(~ 10 nm) CQ-LPs/TAM-NPs(152.8 ± 2.26/153.2 ± 3.05 nm) NNPs/CLT(61.4 ± 2.8 nm, 156.8 ± 2.3 nm, 303.7 ± 1.3 nm)	Sodium taurocholate-induced AP Caerulein and LPS-induce SAP 3% Sodium taurocholate-induce AP	Suppressing the secretion of inflammatory cytokines and regulating the expression of specific apoptotic and anti-apoptotic proteins CQ in combination with TAM syner-gistically promoted iNOS/IDO expression Significantly downregulating the levels of serum amylase and pancreatic myeloperoxidase relevant pro-inflammatory cytokines	Therapy Therapy Therapy	[67] [68] [74]
Oxidative stress and ROS		CAPE-loaded-NL (309 ± 54 nm) RA-EMP (4.703 ± 0.114 nm) NC (82 ± 5.4 nm) NY (159 ± 7.5 nm) Pbzyme (~ 110-nm) MoSe2-PVP NPs (119.39 ± 13.94 nm) MoSe2@PVP NSs (86.278 ± 11.82 nm) Nano-Se (20–60 nm) CA-NPs (50–90 nm)	l-ornithine-induced AP l-arginine-induced AP Caerulein-induced AP Caerulein-induced AP Caerulein-induced AP Caerulein-induced AP Caerulein-induced AP l-arginine-induced AP l-arginine and gamma radiation -induced AP	Modulating Nrf2 and NF-κB Signaling Suppressing the effects of oxidative stress and proinflammatory cytokines Upregulation of Nrf2, SOD1 and NQO1, downregulating the iNOS, p65-NF-κB, Hsp27 and Hsp70 Reducing mitochondrial and ER stress via modulation of Nrf2-NFκB pathway Inhibiting TLRs/NF-κB signaling pathways and scavenging ROS Mimicking CAT, SOD, POD, GPx and eliminating a variety of ROS Mimicking the intrinsic multi-enzyme antioxidant activities of CAT, POD, GPx and SOD to scavenge ROS and RNS Anti-inflammatory, antioxidant and pro-apoptotic actions Down-regulating NLRP3, NF-κB and ASK1/MAPK signal pathways and reducing malondialdehyde and caspase-3 levels	Therapy Therapy Therapy Therapy Therapy Therapy Therapy Therapy Therapy	[83] [84] [87] [88] [17] [89] [90] [91] [92]
Enzymes	Lipase Proteolytic enzymes PLA2	Gd-DTPA-FA(-) BRSNPs (268.65 ± 6.5 nm) LCNPs (89–127 nm) MΦ-NP(L&K) (~ 100 nm)	l-arginine-induced AP l-arginine-induced AP AP Caerulein-induced AP Choline-deficient ethionine (CDE) diet-induced AP	Upon enzymatic hydrolysis by lipase, the fat-soluble Gd-DTPA-FA is converted into a water-soluble Gd-DTPA complex, resulting in the changes of the signal intensities observed with MRI in vitro Inhibiting NF-κB pathway and activating the Nrf2/HO-1 pathway Extending the circulation half-life of the model peptide compound somatostatin Effectively inhibiting PLA2 activity and PLA2-induced pancreatic injury	Diagnosis Therapy Therapy Therapy	[96] [97] [98] [99]
pH		Porous COS@SiO2 nanocomposites (~ 110 nm) Ca-CQ-pDNA-PLGA-NPs (~ 100 nm) FA-SF-NPs (186 nm)	Caerulein and LPS-induced SAP, l-arginine-induced SAP l-arginine-induced AP Biliopancreatic duct ligation- induced AP	Activating the Nrf2 signaling pathway to inhibit oxidative stress and reduce the production of NF-κB and NLRP3 and the release of inflammatory factors Dramatically enhancing gene transfection efficiency showing high targeting efficiency in pancreas Suppressing the inflammation and oxidative stress	Therapy Therapy Therapy	[105] [106] [107]
Multi-targeting		TMSN@PM (~ 142 nm)	l-arginine-induced AP	Scavenging the excess ROS, degrading, and releasing manganese ions for enhanced magnetic resonance imaging	Diagnosis and therapy	[13]

### Microorganisms

AP can be classified as MAP, moderate-to-severe AP (MSAP), and SAP according to the severity [109]. Patients with AP can develop MSAP and SAP, leading to necrotizing pancreatitis (NP), which has a high mortality rate [110]. In the later stage, patients develop intestinal dysfunction and are at risk of the translocation of intestinal flora and secondary infection of necrotic tissue. Most of the bacteria that cause pancreatic tissue necrosis infections are from the intestinal flora [111], mainly including Gram-negative and Gram-positive bacteria. The gut microbiota exists in the inflammatory microenvironment, which is an important mediator during AP and influences the progression of AP.

New imaging strategies have been developed by those researching infectious diseases [112]. For example, Yang’s team introduced an imaging strategy of gold nanoparticles modified by glucose polymer [113], In their study, bacteria ingested nanoparticles through the ATP-binding cassette transporter pathway, and then, under laser irradiation, the nanoparticles aggregated, thereby enhancing the photoacoustic signal. Compared with some optical contrast agents, the nanoparticles can image bacteria in vivo at levels as low as ~ 10^5^ colony-forming units, and have higher sensitivity. Surprisingly, these nanoparticles also have antibacterial activity and an enhanced antibacterial rate. Another photoacoustic contrast agent (AuNPs@P1) not only specifically binds to the cell surface of *Staphylococcus aureus* in the infected area through an active targeting mechanism, but also induces the targeted aggregation of gold nanoparticles at the site of infection through bacterial overexpression of collagenase IV, which significantly enhances the photoacoustic signal [114]. Compared with traditional AuNPs, AuNPs@P1 has higher sensitivity and specificity.

On the treatment side, the regulation or suppression of intestinal flora may be an effective treatment for AP. Antibiotics are now often used to prevent infection in pancreatic necrosis. However, the long-term use of antibiotics increases the level of bacterial resistance and the incidence of fungal infections [115]. Nanomaterials could be developed to replace biocides and provide new antimicrobial strategies for the treatment of infectious diseases due to its excellent anti-infection ability [116, 117]. It has been reported that metal-based nanomaterials usually have broad-spectrum antibacterial properties with no drug resistance. Silver and photothermal therapy (PTT) are highly effective in killing drug-resistant bacteria. Our group combined these two therapies to develop a bacterial-targeted therapeutic platform (Ag^+^-GCS-PDA@GNRs) based on polydopamine (PDA)-encapsulated gold nanorods (GNRs) to exert synergistic antibacterial activity [118]. Ag^+^-GCS-PDA@GNRs release Ag^+^ under acidic conditions and deliver it to abscess environment to destroy the cell membrane of bacteria and reduce their heat resistance. On the other hand, combined PPT treatment not only kills bacterial cells but also triggers the release of more Ag^+^, exhibiting higher bactericidal efficiency. Yu and colleagues developed a new strategy to eradicate drug-resistant bacteria by inducing the production of alkyl radicals [119]. An antimicrobial depot (AIBI-GCS-PDA@CG) was synthesized by modifying polydopamine-coated carboxyl graphene with glycol chitosan (GCS) and utilizing an initiator of 2,2-azobis[2-(2-imidazolin-2-yl)propane]dihydrochloride (AIBI) as a source of free radicals. Under the conditions of acidic pus and hypoxia at the infected site, AIBI-GCS-PDA@CG decomposed into alkyl radical by NIR radiation, which destroyed bacterial DNA and led to the death of bacteria. Additionally, it has been demonstrated that AIBI-GCS-PDA@CG has an equivalent therapeutic effect on bacteria under normoxic conditions. Based on the role of macrophage membranes in bacterial recognition, Wang et al. designed a macrophage-membrane-coated gold nanocage that can target bacteria more effectively by improving bacterial adhesion and retaining bacteria at the site of infection [120].

With disease aggravation, AP progresses to NP. Infection is a characteristic of patients with NP. Nanotechnology can be applied to exploit unique targets of the infectious microenvironment to design nanoparticles that target the site of infection, improving diagnostic rates and therapeutic efficacy. Although many studies have reported the use of nanomaterials for the treatment and diagnosis of infectious diseases, few have been reported for acute NP, which has prompted us to shift our research plans toward the use of nanotechnology for the diagnosis and anti-infective treatment of AP.

### Hypoxia

Most inflammatory diseases are characterized by hypoxia, and AP is no exception. The inflammatory response in diseased tissues increases metabolic activity and leads to hypoxia, which then activates HIF-1α [121]. It has been reported that HIF-1α is involved in the histopathological progression of AP [122]. Knockdown of HIF-1α reduces the production of ROS to attenuate the necrosis of pancreatic follicular cells [123]. AP with pulmonary dysfunction further aggravates hypoxia, leading to hypoxemia and acute respiratory distress syndrome [124]. The anoxic nature of AP offers the possibility to design anoxia-responsive nanoplatforms. Currently, hypoxia-responsive nanomaterials are widely used in the treatment and diagnosis of tumors. It is known that nitroreductase (NTR) is highly expressed in hypoxic regions. Based on this, Zheng et al. reported a near-infrared off–on fluorescence probe (Cy-NO_2_) [125]. Cy-NO_2_ was found to be hypersensitive and highly selective for rapid response to NTR, which is ideal in models of cerebral ischemia and deep vein thrombosis. Zhang et al. proposed hypoxia-responsive nanocarriers (CPs-CPT-Ce6 NPs) combined with photodynamic therapy (PDT) as a strategy to treat tumors [126]. Upon irradiation with a laser, CPs-CPT-Ce6 NPs release ROS, which aggravate tissue hypoxia and lead to the release of camptothecin (CPT) from conjugated polymers (CPs) loaded with photosensitizers and chemotherapeutic agents, enhancing the therapeutic effect. Combined treatment of CPs-CPT-Ce6 NPs with PDT exerts a synergistic effect to enhance the potency of killing PDT-resistant tumor cells. Hypoxia-responsive nanoplatforms that combine diagnosis and treatment have also been developed. For example, Zhou and colleagues constructed an azo-based hypoxia-responsive theranostic agent (AzP1) [127]. Under hypoxic conditions, cleavage of the azo bond in AzP1 releases the drug (SN-38) with enhanced cytotoxicity. Additionally, SN-38 exhibits fluorescence enhancement at λ_Em_ = 560 nm and can be used for inflammation-specific imaging. AzP1 is an excellent theranostic system that combines hypoxia response therapy and imaging technology.

Controlling hypoxia may be one way of alleviating the symptoms of AP. In a clinical context, physicians mainly depend on oxygenation to relieve patients’ hypoxia. However, the potential toxicity of an excessive oxygen supply hinders its clinical application [128]. Nanotechnology offers a new approach to oxygen supply. To date, researchers have adopted different strategies to overcome hypoxia and improve the treatment effect. One strategy is to prepare O_2_ carriers using smart nanomaterials to transport molecular oxygen directly to the hypoxic site. Shi et al. used hemoglobin (Hb) to synthesize a multifunctional nanoprobe (Gd@Hb^Ce6−PEG^) based on Gd-based nanostructures [129]. This probe can be loaded with the photosensitizer chlorine e6 (Ce6) and oxygen to alleviate the hypoxic environment of the tumor. Under laser irradiation, Gd@Hb^Ce6−PEG^ produces oxygen and kills tumor cells. Experiments have shown that Gd@Hb^Ce6−PEG^ has excellent oxygen-carrying capacity, which can enhance the therapeutic effect of PDT. Another strategy is to generate O_2_ in situ by H_2_O_2_ based on the characteristics of the tumor microenvironment. In recent years, various nanoparticle-based catalysts/enzymes have been constructed to catalyze the decomposition of H_2_O_2_ to generate O_2_ to ameliorate tumor hypoxia. For example, Wang and colleagues designed a multifunctional mesoporous nanoenzyme that reacts with endogenous H_2_O_2_ to produce O_2_ and successfully ameliorate tumor hypoxia [130]. Moreover, Liu et al. created a core–shell nanosystem that uses catalase to catalyze the decomposition of H_2_O_2_ into O_2_ to alleviate inflammatory hypoxia and inhibit HIF-1α expression [131]. However, there is no particularly precise method for the real-time dynamic monitoring and control of local oxygen concentration.

### Multitargeting strategies

In the inflammatory microenvironment in AP, excessive digestive enzymes, H^+^, inflammatory cells, and ROS are major parts of the inflammatory response, which can exacerbate the progression of AP through various mechanisms. Therefore, the use of a single index for diagnosis and treatment has limited efficacy. The coexistence of these inflammation-associated substances provides additional opportunities for the diagnosis and treatment of AP. In recent years, multitargeted drug delivery systems have been developed. In contrast, dual-targeted drug delivery systems can better utilize the microenvironment to achieve efficient drug delivery. Dai et al. constructed a nanosystem with self-amplified drug release for synergistic oxidation-chemotherapy [132]. The system is not only pH-sensitive, enhancing the cellular uptake of drugs by shifting surface charge, but also responds to ROS to release β-lapachone and cephaeline, overcoming multidrug resistance at tumor sites and promoting apoptosis in tumor cells. In another study, Gou et al. designed SF nanoparticles (CS-CUR-NP) surface-functionalized (SF) with chondroitin sulfate (CS). Upon stimulation by pH and ROS, SF can release CUR on demand. Moreover, CS-CUR-NP has the ability to target macrophages and can release drugs at inflammatory sites for the treatment of ulcerative colitis [133]. Unexpectedly, nanoparticles for AP therapy and diagnosis have also been developed. Our group developed a nanotheranostic agent (TMSN@PM) with slight acidic and excessive ROS stimulation. TMSN@PM can be used for MRI and therapy of AP. The development of TMSN@PM provides a precedent for combining diagnostic and therapeutic applications in AP, and it is believed that more multifunctional nanoplatforms will be applied to AP in the future with the joint efforts of researchers.

**Fig. 6:**
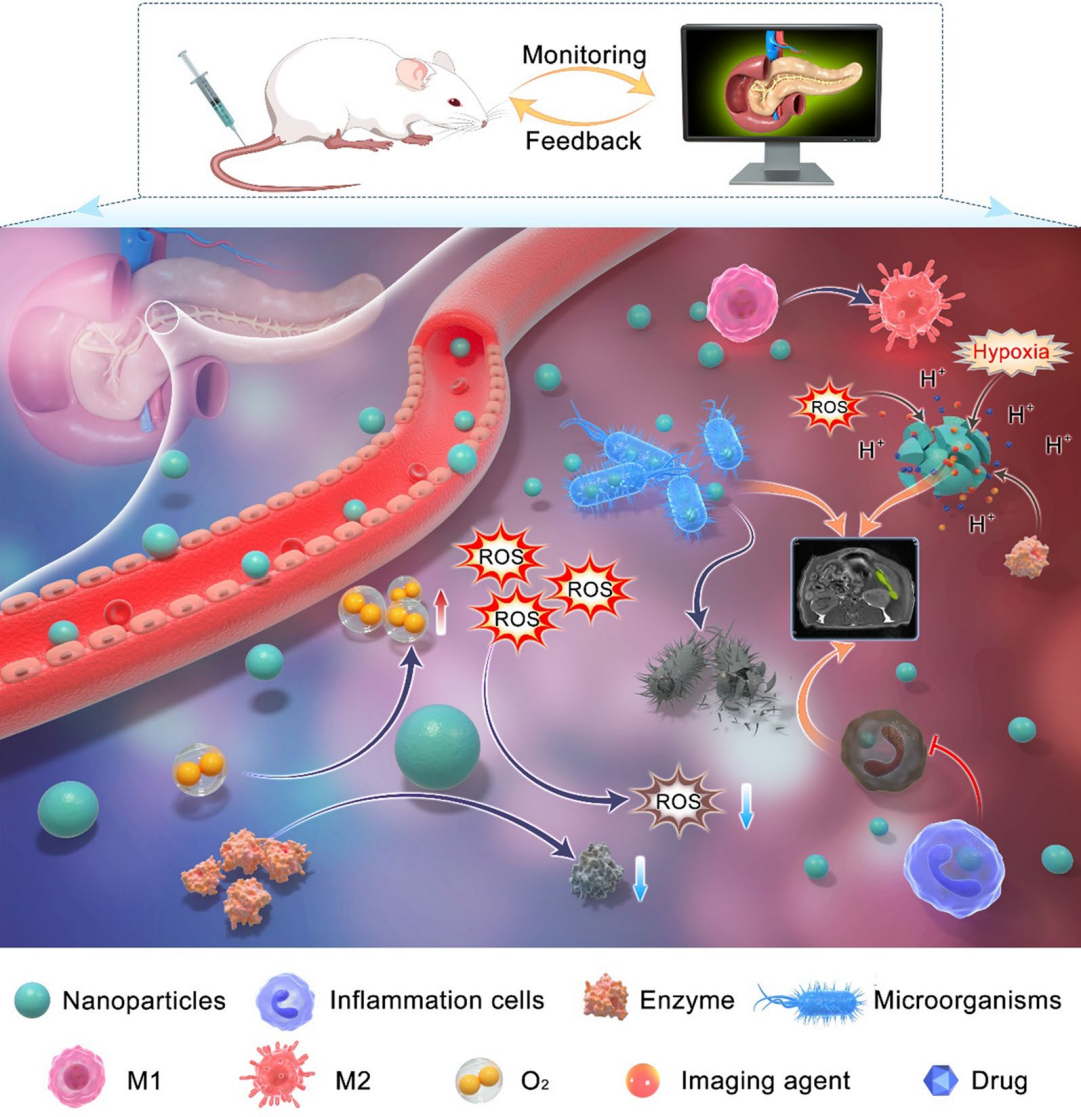
Schematic diagram of different modes of interaction between nanoparticles and targets in the inflammatory microenvironment. Nanoparticles are released from blood vessels into the pancreatic inflammatory microenvironment, which not only react with reduced oxygen and excess enzymes, ROS, and H^+^ to release imaging agents and drugs for diagnostic and therapeutic purposes, but also act directly on these targets to reduce ROS and enzymes and increase oxygen. Additionally, inflammatory cells and bacteria can phagocytose nanoparticles for imaging and exert anti-inflammatory and antibacterial effects. Meanwhile, M1 macrophages were shown to be regulated to M2 macrophages by the action of nanoparticles and changes in the microenvironment. The application of nanotechnology can monitor and reduce the severity of acute pancreatitis

The complex microenvironmental changes in AP increase the difficulty of diagnosis and treatment, but also provide more targets, as shown in Fig. 6. Compared with a single targeting drug, regulating two or more targets simultaneously improves the effect. Therefore, finding more targets and designing multitargeting nanocarriers are expected to lead to better strategies for the diagnosis and treatment of AP.

## Conclusions and outlooks

AP is a complex inflammatory disease associated with multiple mechanisms, such as Ca^2+^ overload, mitochondrial dysfunction, trypsinogen activation, impaired autophagy, and ER stress. In the early stage of AP, mild damage to pancreatic tissue occurs, but as the disease progresses it can lead to SIRS and MODS in severe and life-threatening cases. Therefore, early diagnosis and treatment are crucial for patients with AP.

In terms of diagnosis, the development of nanotechnology has addressed the limitations of conventional medical diagnosis and treatment. On the one hand, nanomaterials with a highly selective bioresponse can improve sensitivity and reduce response time, thus increasing the efficiency of various detection techniques [95, 134]. On the other hand, various biosensors and nanoprobes have been developed as indicators of the inflammatory microenvironment. Nanomaterials can enhance their performance and characteristics through surface modification, accumulating in organs in a targeted way and increasing the signal intensity [135]. Therefore, nanotechnology has been widely used in the diagnosis of AP by improving the efficiency of pancreatic enzyme detection and pancreatic tissue imaging, improving the diagnostic rate of AP.

In terms of treatment, in recent years a series of nanodrugs and nanocarrier systems have been developed to restore the damage of AP, which has solved the shortcomings of poor solubility and bioavailability of conventional drugs and improved the efficiency of drug delivery [38]. Furthermore, therapeutic nanomaterials targeting constituents of the microenvironment have been applied to treat AP, exerting anti-inflammatory, antioxidant, and antiapoptotic effects. The inflammatory microenvironment forms when AP occurs, so carriers that target abnormal biochemical indicators (H^+^, ROS, abundant digestive enzymes, etc.) in the microenvironment can be designed by applying surface functionalization technologies and stimulus-responsive materials to achieve targeted drug delivery to pancreatic tissues and related damaged organs and prolong the residence time at the site of inflammation. Additionally, through the construction of responsive drug delivery systems, the substances overexpressed in the microenvironment are consumed to produce synergistic effects and improve the therapeutic efficacy. Therefore, targeting corresponding indicators in the microenvironment for drug therapy is an attractive strategy and raises the possibility of developing effective treatments of various inflammatory diseases.

Nanotechnology has achieved excellent results in the diagnosis and treatment of AP by targeting potential targets in the inflammatory microenvironment, but there are still many challenges and limitations. Firstly, due to the anatomical features of the pancreas, there exists a unique BPB composed of the capillary endothelial cell layer around the glandular follicles, the basement membrane layer and other structures, which prevents pancreas from pathogenic microorganisms infecting but creates a certain obstacle to the delivery of drugs into the pancreas at the same time, resulting in a limited number of types of drugs that can effectively cross the BPB as well as a low effective concentration and utilization rate [72, 136]. Currently, nanocarriers targeting the inflammatory microenvironment are limited to a single type, resulting in low delivery efficiency. In the future, the advantages of different carriers can be combined to develop carriers with higher delivery efficiency and biosafety. Secondly, the pathogenesis of AP has not been fully clarified, so there are not enough specific targets for AP, resulting in fewer nanodrugs that can specifically enter the pancreatic inflammatory microenvironment. At present, most nanodrugs passively target inflammatory lesions through the ELVIS effect to increase the drug concentration, but there is an off-target effect, which leads to low efficiency of nanodrug delivery [74, 107]. There is an urgent need to develop more active targeted nanodrugs combined with target molecules, instead of passive targeting, and to improve the delivery efficiency. Recently, the emergence of genomics, protein genomics and metabonomics has made it possible to find more specific markers of pancreatitis, thus improving the diagnosis rate and treatment efficiency.

The development and application of functional nanomaterials targeting various potential targets in the inflammatory microenvironment is a future trend in the early diagnosis and treatment of AP, but there are still many concerns that have led to the fact that nanomaterials are not yet widely used in the clinic, and one of the most important issue is the biosafety of nanomaterials. On the one hand, we still know little about the risks and their potential threats of nanomaterials, and the fact of lacking regulatory guidance and uniform standards for the toxicological assessment of nanomaterial-based drug delivery systems worsen this situation [137, 138]. On the other hand, most of the experiments conducted to date have been based on animal models, however, it is difficult for animal models to simulate the absorption, distribution, metabolism and excretion of nanomaterials in the human body as well as their effects on organs and tissues due to the complexity of the human immune system in terms of drug metabolism. Moreover, there are many common methods to establish AP models [97, 99, 107], and forms of AP caused by various different factors have their own specific characteristics. Researchers need to know the pathophysiology and limitations of each model in order to choose the appropriate model according to their own experimental needs. The technology for preparing AP models is not fully mature, and it is difficult for animal models to simulate the pathogenesis of human AP due to the diversity and complexity of its causes. Therefore, it is necessary to establish a unified scientific evaluation system, improve animal models, evaluation indexes and testing methods, and vigorously develop nanotoxicology, so as to promote the clinical translation of nanomaterials.

With the joint efforts of researchers in the future, multicenter and large-scale clinical trials can be realized. Additionally, with continuous technological development, artificial intelligence, big data analysis, 3D printing technology, and other fields have emerged, with which nanotechnology can be combined to generate new innovations and improve the ability to diagnose and treat human diseases, thus bringing a new era in the field of medicine.

## Data Availability

Not applicable.

## Abbreviations

AP: Acute pancreatitis
ROS: Reactive oxygen species
MAP: Moderate acute pancreatitis
SAP: Severe acute pancreatitis
ER: Endoplasmic reticulum
SIRS: Systemic inflammatory response syndrome
MODS: Multiple organ dysfunction syndrome
HCQ: Hydroxychloroquine
MRI: Magnetic resonance imaging
HIF-1α: Hypoxia-inducible factor-1α
NF-κB: Nuclear factor-kappa B
TLR: Toll-like receptor
ELVIS effect: The effect of extravasation through leaky vasculature and subsequent inflammatory cell-mediated sequestration
CUR: Curcumin
SPION: Superparamagnetic iron oxide nanoparticles
NIRF: Near-infrared fluorescence
SPIO: Superparamagnetic iron oxide
ATP: Adenosine triphosphate
NE: Neutrophil elastase
MMSNs: Magnetic mesoporous silica nanoparticles
NETs: Neutrophil extracellular traps
BMSCs: Bone marrow-derived mesenchymal stem cells
RNS: Reactive nitrogen species
OA: Osteoarthritis
DEX: Dexamethasone
TKCP: Thioketal linkers and cartilage-targeting peptide
AIE: Aggregation-induced emission
CAPE-loaded NL: CAPE-loaded nanoliposomes
EMP: Empagliflozin
NY: Nanoyttria
PBzyme: Prussian blue nanoenzyme
NSs: Nanosheets
CA-NPs: Cinnamic acid nanoparticles
SST: Somatostatin
PLA2: Phospholipase A2
MnO_2_: Manganese dioxide
CQ: Chloroquine diphosphate
SF: Silk fibroin
FA: Ferulic acid
Fe-Ce-MSN: Fe/Ce-doped mesoporous silica nanoparticles
TNF-α: Tumor necrosis factor-α
IL-1β: Interleukin-1β
MSAP: Moderate-to-severe acute pancreatitis
NP: Necrotizing pancreatitis
PTT: Photothermal therapy
GCS: Glycol chitosan
GNRs: Gold nanorods
AIBI: 2,2-Azobis[2-(2-imidazolin-2-yl)propane]dihydrochloride
PDA: Polydopamine
NTR: Nitroreductase
PDT: Photodynamic therapy
CPs: Conjugated polymers
CPT: Camptothecin
Hb: Hemoglobin
Ce6: Chlorine e6
CS: Chondroitin sulfate
SF: Surface-functionalized
