# Correction: Microenvironment of pancreatic inflammation: calling for nanotechnology for diagnosis and treatment

**DOI:** 10.1186/s12951-023-02232-3

**Published:** 2024-01-23

**Authors:** Lu Liu, Yiqing Zhang, Xinghui Li, Jun Deng

**Affiliations:** 1 Medical Imaging Key Laboratory of Sichuan Province, Department of Radiology, Affiliated Hospital of North Sichuan Medical College, 1 South Maoyuan Street, Nanchong, 637001 China; 2 Institute of Burn Research Southwest Hospital State Key Lab of Trauma Burn and Combined Injury Chongqing Key Laboratory for Disease Proteomics Army Medical University, Chongqing, 400038 China; 3 Research Center for Tissue Repair and Regeneration Affiliated to the Medical Innovation Research Division and the 4th Medical Center of Chinese PLA General Hospita, PLA Medical College, 28 Fu Xing Road, Beijing, 100853 China

**Correction: Journal of Nanobiotechnology (2023) 21:443** 10.1186/s12951-023-02200-x

Following publication of the original article [1], the cells in Table 1 were not formatted and aligned correctly.

**Table 1:** Nanotechnology-based strategies for the diagnosis and treatment of acute pancreatitis

Category	Indicators	Nanoagents (Size)	Animal models	Mechanisms	Modes	Refs.
Inflammatory cells	Macrophage	Lip-DTPA@AuNP(17.2 ± 2.1 nm) M-Gd-NL (120.2 ± 8.5 nm)	Caerulein and LPS-induced AP l-Arginine-induced AP	Gd (III) contrast agents loading of AuNPs and localization to pancreatic tissue for MR imaging	Diagnosis	[47]
		Gd-DTPA-Cy5.5-PsLmAb (50 mm) CO-HbV (~ 280 nm)	Caerulein-induced AP, l-A-arginine-induced AP –	Targeting macrophages and increasing T1 Imaging ability	Diagnosis	[48]
		G4.5-COOH, G5-OH (5 nm) SPIO-clodronate-liposomes (100–200 nm) MU (175 nm)	Caerulein-induced AP	P-selectin-targeted MR/NIRF bimodal imaging improves spatial resolution and sensitivity.	Diagnosis	[49]
			Sodium taurocholate-induced SAP Caerulein-induced AP	Targeting macrophages and polarizing macrophages toward an M2-like phenotype	Therapy	[56]
				Inhibition of NF-κB nuclear translocation in macrophages and a reduction in inflammatory cells	Therapy	[57]
				Selectively inducing macrophage apoptosis and reducing the release of inflammatory mediators	Diagnosis and therapy	[59]
				Significantly inhibiting the secretion of pro-inflammatory cytokines TNF-α and IL-6 by macrophages	Therapy	[60]
	Neutrophil	tFNAs (~ 10 nm)	Sodium taurocholate-induced AP	Suppressing the secretion of inflammatory cytokines and regulating the expression of specific apoptotic and anti-apoptotic proteins	Therapy	[67]
		CQ-LPs/TAM-NPs(152.8 ± 2.26/153.2 ± 3.05 nm)	Caerulein and LPS-induce SAP	CQ in combination with TAM syner-gistically promoted iNOS/IDO expression	Therapy	[68]
		NNPs/CLT (61.4 ± 2.8 nm、156.8 ± 2.3 nm、303.7 ± 1.3 nm)	3% Sodium taurocholate-induce AP	Significantly downregulating the levels of serum amylase and pancreatic myeloperoxidase elevant pro-inflammatory cytokines	Therapy	[74]
Oxidative stress and ROS		CAPE-loaded-NL (309 ± 54 nm) RA-EMP (4.703 ± 0.114 nm)	l-Ornithine-induced AP	Modulating Nrf2 and NF-κB Signaling	Therapy	[83]
			l-Arginine-induced AP	Suppressing the effects of oxidative stress and proinflammatory cytokines	Therapy	[84]
		NC (82 ± 5.4 nm)	Caerulein-induced AP	Upregulation of Nrf2, SOD1 and NQO1, downregulating the iNOS, p65-NF-κB, Hsp27 and Hsp70	Therapy	[87]
		NY (159 ± 7.5 nm)	Caerulein-induced AP	Reducing mitochondrial and ER stress via modulation of Nrf2-NFκB pathway	Therapy	[88]
		Pbzyme (~ 110-nm)	Caerulein-induced AP	Inhibiting TLRs/NF-κB signaling pathways and scavenging ROS	Therapy	[17]
		MoSe2-PVP NPs (119.39 ± 13.94 nm) MoSe2@PVP NSs (86.278 ± 11.82 nm)	Caerulein-induced AP	Mimicking CAT, SOD, POD, GPx and eliminating a variety of ROS	Therapy	[89]
			Caerulein-induced AP	Mimicking the intrinsic multi-enzyme antioxidant activities of CAT, POD, GPx and SOD to scavenge ROS and RNS	Therapy	[90]
		Nano-Se (20–60 nm)	l-Arginine-induced AP	Anti-inflammatory, antioxidant and pro-apoptotic actions	Therapy	[91]
		CA-NPs (50–90 nm)	l-Arginine and gamma radiation-induced AP	Down-regulating NLRP3, NF-κB and ASK1/MAPK signal pathways and reducing malondialdehyde and caspase-3 levels.	Therapy	[92]
Enzymes	Lipase	Gd-DTPA-FA (−)	l-Arginine-induced AP	Upon enzymatic hydrolysis by lipase, the fat-soluble Gd-DTPA-FA is converted into a water-soluble Gd-DTPA complex, resulting in the changes of the signal intensities observed with MRI in vitro	Diagnosis	[96]
	Proteolytic enzymes	BRSNPs (268.65 ± 6.5 nm)	l-Arginine-induced AP	Inhibiting NF-κB pathway and activating the Nrf2/HO-1 pathway	Therapy	[97]
		LCNPs (89–127 nm)	AP	Extending the circulation half-life of the model peptide compound somatostatin	Therapy	[98]
	PLA2	MΦ-NP (L&K) (~ 100 nm)	Caerulein-induced AP Choline-deficient ethionine (CDE) diet-induced AP	Effectively inhibiting PLA2 activity and PLA2-induced pancreatic injury	Therapy	[99]
pH		Porous COS@SiO_2_ nanocomposites (~ 110 nm)	Caerulein and LPS-induced SAP, l-arginine-induced SAP	Activating the Nrf2 signaling pathway to inhibit oxidative stress and reduce the production of NF-κB and NLRP3 and the release of inflammatory factors	Therapy	[105]
			l-Arginine-induced AP	Dramatically enhancing gene transfection efficiency showing high targeting efficiency in pancreas	Therapy	[106]
		Ca-CQ-pDNA-PLGA-NPs (~ 100 nm) FA-SF-NPs (186 nm)	Biliopancreatic duct ligation- induced AP	Suppressing the inflammation and oxidative stress	Therapy	[107]
Multi-targeting		TMSN@PM (~ 142 nm)	l-Arginine-induced AP	Scavenging the excess ROS, degrading, and releasing manganese ions for enhanced magnetic resonance imaging	Diagnosis and therapy	[13]

The correct Table 1 is included in this Correction, and the original article has been corrected.
